# Characterization of cross-tissue genetic-epigenetic effects and their patterns in schizophrenia

**DOI:** 10.1186/s13073-018-0519-4

**Published:** 2018-02-26

**Authors:** Dongdong Lin, Jiayu Chen, Nora Perrone-Bizzozero, Juan R. Bustillo, Yuhui Du, Vince D. Calhoun, Jingyu Liu

**Affiliations:** 1 The Mind Research Network and Lovelace Biomedical and Environmental Research Institute, Albuquerque, NM 87106 USA; 2 Department of Neurosciences, University of New Mexico, Albuquerque, NM 87131 USA; 3 Department of Electrical and Computer Engineering, University of New Mexico, Albuquerque, NM 87131 USA; 4 Department of Psychiatry, University of New Mexico, Albuquerque, NM 87131 USA

**Keywords:** Genetic, DNA methylation, meQTLs, eQTL, Cross-tissue, Schizophrenia, Co-methylation network

## Abstract

**Background:**

One of the major challenges in current psychiatric epigenetic studies is the tissue specificity of epigenetic changes since access to brain samples is limited. Peripheral tissues have been studied as surrogates but the knowledge of cross-tissue genetic-epigenetic characteristics remains largely unknown. In this work, we conducted a comprehensive investigation of genetic influence on DNA methylation across brain and peripheral tissues with the aim to characterize cross-tissue genetic-epigenetic effects and their roles in the pathophysiology of psychiatric disorders.

**Methods:**

Genome-wide methylation quantitative trait loci (meQTLs) from brain prefrontal cortex, whole blood, and saliva were identified separately and compared. Focusing on *cis*-acting effects, we tested the enrichment of cross-tissue meQTLs among cross-tissue expression QTLs and genetic risk loci of various diseases, including major psychiatric disorders. CpGs targeted by cross-tissue meQTLs were also tested for genomic distribution and functional enrichment as well as their contribution to methylation correlation across tissues. Finally, a consensus co-methylation network analysis on the cross-tissue meQTL targeted CpGs was performed on data of the three tissues collected from schizophrenia patients and controls.

**Results:**

We found a significant overlap of *cis* meQTLs (45–73 %) and targeted CpG sites (31–68 %) among tissues. The majority of cross-tissue meQTLs showed consistent signs of *cis*-acting effects across tissues. They were significantly enriched in genetic risk loci of various diseases, especially schizophrenia, and also enriched in cross-tissue expression QTLs. Compared to CpG sites not targeted by any meQTLs, cross-tissue targeted CpGs were more distributed in CpG island shores and enhancer regions, and more likely had strong correlation with methylation levels across tissues. The targeted CpGs were also annotated to genes enriched in multiple psychiatric disorders and neurodevelopment-related pathways. Finally, we identified one co-methylation network shared between brain and blood showing significant schizophrenia association (*p* = 5.5 × 10^−6^).

**Conclusions:**

Our results demonstrate prevalent cross-tissue meQTL effects and their contribution to the correlation of CpG methylation across tissues, while at the same time a large portion of meQTLs show tissue-specific characteristics, especially in brain. Significant enrichment of cross-tissue meQTLs in expression QTLs and genetic risk loci of schizophrenia suggests the potential of these cross-tissue meQTLs for studying the genetic effect on schizophrenia. The study provides compelling motivation for a well-designed experiment to further validate the use of surrogate tissues in the study of psychiatric disorders.

**Electronic supplementary material:**

The online version of this article (10.1186/s13073-018-0519-4) contains supplementary material, which is available to authorized users.

## Background

DNA methylation, as one of the most broadly studied epigenetic modifications, can influence the way genes are structured and expressed, and ultimately affect cell function without modifying the underlying sequence. Increasing evidence has shown that methylation can modulate genetic risks and environmental effects in neuron cell differentiation, cell development, and neurogenesis and plays a vital role in pathogenesis of mental disorders [1–4]. CpGs that undergo substantial methylation changes in early developmental stages have been found to be enriched in psychiatric disorders [5]. A recent study reviewed 33 studies on peripheral tissue DNA methylation in schizophrenia (SZ) and/or bipolar disorder (BIP) and found moderate evidence of consistent differential methylation at some genetic loci across studies [6]. Particularly for SZ, specific CpG methylation patterns have been related to SZ-positive symptoms [7], disease onset [8], and cognitive deficit [9] in adulthood.

DNA methylation can be influenced by underlying sequence variants. For example, genotype variation or specific alleles of a locus (i.e., single nucleotide polymorphisms (SNPs)) can influence CpG methylation state, termed methylation quantitative trait loci (meQTL) effect [5, 10]. The effects of most meQTLs are *cis*, targeting proximal CpG sites, while some are *trans*, targeting distal CpG sites. A number of studies have reported meQTL–CpG relationships in human cell lines [11, 12], peripheral tissues [13, 14], and the brain [15]. These findings indicate that meQTLs are more likely to reside at regulatory elements than expected by chance and coincide with changes in transcription factor binding, chromatin conformation, gene expression, RNA splicing, and, potentially, disease risk [12, 16, 17].

meQTLs have also gained increasing interest in recent psychiatric epigenetic studies at the early neurodevelopment stages and in adulthood [4, 16]. meQTLs from postmortem brain and peripheral tissues have shown significant enrichment for susceptible genetic variants of autism spectrum disorder (ASD) [18], BIP [19], and SZ [13, 20]. Two recent landmark studies comprehensively explored the role of DNA methylation and meQTLs in brain development as well as their relationship with SZ [1, 5]. They found a large overlap of meQTLs between fetal and adult brain tissues and their significant enrichment in SZ risk loci. In addition, these meQTLs were also significantly enriched in expression QTLs (eQTLs), suggesting the potential of meQTLs to exert their effect through methylation, impacting gene expression and leading to further changes of cell or organ function and disease.

However, tissue specificity of DNA methylation poses a challenge for studying methylation in psychiatric disorders due to very limited access to brain samples [21]. Several studies have attempted to compare methylation patterns among brain and peripheral tissues (e.g., blood and saliva) and identified a small proportion (2–7 %) of CpG sites with highly correlated methylation patterns among tissues [22–24], for which Hannon et al. [25] found an underlying genetic contribution. A recent study compared meQTLs across tissue types and their enrichment in ASD genomic risk [18], but the effects of meQTLs may also be susceptible to tissue specificity, similar to tissue-specific eQTL effects as reported by the GTEx project [26]. Monozygotic and dizygotic twin studies have shown variable heritability (12–30 %) of DNA methylation across different tissues [27–29]. Tissue-specific meQTLs with varying effects by tissue type or cell line have also been reported [10, 30]. Current knowledge of meQTLs across tissues as well as their role in regulating methylation and gene expression, particularly in the context of psychiatric disorders, is very limited.

To better understand meQTLs and their targeted CpGs across tissue types, in this work we attempted to leverage large-scale genomic and DNA methylation data from brain and peripheral tissues (blood and saliva) to explore the following questions: 1) whether meQTLs from different tissues are highly consistent in terms of regulating *cis*-CpGs; 2) how cross-tissue meQTL-targeted CpGs are distributed across the genome and among gene functional annotations; 3) whether cross-tissue meQTLs relate to susceptibility to psychiatric disorders and are enriched for eQTLs; 4) whether cross-tissue meQTLs contribute to the methylation level correlation of targeted CpGs across tissues; and 5) whether cross-tissue meQTL-targeted CpGs demonstrate consensus methylation networks across tissue types. This work is expected to enrich our understanding of cross-tissue meQTL effects in diseases and provide more evidence to guide future investigations of psychiatric disorders by integrating genetic, epigenetic, and gene expression data in diverse tissue types.

## Methods

We compared meQTLs and CpGs from brain, blood, and saliva. Genotype and methylation data from saliva were collected from the Center for Biomedical Research Excellence study [31] and the Glutamate and Outcome in Schizophrenia study [32]. meQTL data from brain and blood were obtained from two other published studies [1, 13].

### Saliva samples

Patients with a diagnosis of SZ or schizoaffective disorder between 18 and 65 years of age were recruited. Age-matched controls were recruited from the same geographic location. Detailed inclusion and exclusion criteria have been described elsewhere [14]. Saliva samples from 99 SZ patients and 98 controls were collected for genotyping and methylation detection.

### Saliva DNA genotyping

Genotyping for DNA from saliva was performed using two assays: Illumina Infinium Human Omni1-Quad assay and Infinium Omni5 assay. Both datasets were quality controlled separately (due to different arrays) using PLINK software (http://zzz.bwh.harvard.edu/plink/) as introduced in [14], mainly including removal of subjects (missing rate > 10 %) and SNPs (genotyping rate < 90 %, Hardy-Weinberg equilibrium < 10^−6^ or minor allele frequency (MAF) < 0.01). Missing value imputation was performed using the 1000 Genomes reference panel phase 1, version 3 and the software IMPUTE2 [33]. Loci with a high imputation quality score (> 0.9) from the two datasets were merged, resulting in 10,513,590 loci. After further quality control (missing rate > 1 %, MAF < 0.05), 3,622,550 loci were left for analysis. We adjusted for the population structure by using the first three principal components (PCs) of the genotype matrix.

### Saliva DNA methylation

DNA methylation was measured using the Infinium MethylationEPIC assay, covering 866,836 CpG sites. A series of quality control steps were performed using the R package ‘minfi’ [34] as applied in [14]. Both methylated and unmethylated signals were normalized using the quantile-based normalization method on each site. Beta values were used in subsequent preprocessing, including removing 1) CpGs coinciding with SNPs or at single nucleotide extensions [35]; 2) CpGs with non-specific probes [36]; 3) CpGs with more than 1 % missing values (methylation values with detection *p* > 0.05 were treated as missing values); and 4) CpGs on sex chromosomes. The remaining missing beta values were further imputed using the average of each CpG as applied in [37] and some other microarray studies [38]. After preprocessing, 363,366 CpGs were kept. Batch effects were then corrected for each CpG using a parametric Bayes framework implemented in the ‘combat’ function [39] in the R package ‘SVA’ [40] prior to meQTL analysis. Cell type proportions in saliva samples were estimated by the algorithm described by Houseman et al. [41] using methylation data from buccal epithelial cells (GSE46573) and other leukocyte cell types from the minfi package as the reference.

### meQTL detection

#### Saliva meQTLs

The association analysis between 3,622,550 SNPs and 363,366 CpGs was performed by a linear additive regression model using Matrix eQTL software [42]. The association tests for SNP–CpG pairs were restricted to distances within 20 kbp to focus on *cis*-acting genetic effects. The covariates age, sex, cell type proportion, diagnosis, and top three ancestry-related PCs from merged genotypes were adjusted in an association analysis. We identified 825,405 autosomal SNP–CpG pairs with significance *p* ≤ 1 × 10^−5^.

#### Brain meQTLs

Brain meQTLs were derived from prefrontal cortex (dorsolateral prefrontal cortex, BA46/9) of 258 healthy subjects (aged > 13 years) in a published study [1]. As described by Jaffe et al. [1], 7,426,085 SNP genotypes and 477,636 CpG beta values after quantile-based normalization were used for meQTL analysis using a linear additive regression model in Matrix eQTL, resulting in 4,107,214 significant, false discovery rate (FDR)-corrected SNP–CpG association pairs (within 20 kbp, *p* < 8.6 × 10^−4^) after controlling for covariates related to ancestry (first five multidimensional scaling components) and global epigenetic variation (first 11 PCs).

#### Blood meQTLs

Blood meQTLs were obtained from a longitudinal study [13]. The authors rank-normalized methylation levels of 395,625 CpGs and combined them with 8,074,398 SNP loci for meQTL analysis by controlling for the covariates age, sex, batch, cell count, and top ten ancestry-related PCs, resulting in 5,317,173 SNP–CpG pairs (*p* < 1 × 10^−7^ in at least one age group). Although their meQTL analysis shows highly stable genetic effects on methylation level across lifespan, to best match the age distribution of brain and saliva studies, we chose the meQTL results derived from the peripheral blood of 837 adolescents (age 17.14 ± 1.01 years) for comparative analysis.

To make the meQTL results comparable across tissues, we restricted our analyses by: 1) focusing on the SNPs and CpGs shared among the involved datasets (annotated by 1000 Genomes Project phase 1, version 3 reference panel) and from autosomal chromosomes; 2) filtering out CpGs either coinciding with SNPs or at single base extensions [35] or probed with non-specificity [36]; 3) considering significant *cis* meQTL effects only when SNP–CpG distance < 20 kbp and association *p* ≤ 1 × 10^−5^, comparable to the thresholds applied in other meQTL studies using Methylation 450K chips (FDR < 0.01) [1, 43].

### meQTLs and targeted CpGs overlap across tissue types

SNPs and CpGs were matched by their chromosome positions across tissue types. For the common SNPs and CpGs in each pair of tissues, we evaluated the percentages of SNPs and CpGs showing *cis*-meQTL effects in each tissue and their overlap between tissues. meQTL alleles were also matched across tissues (flip strand and switch coding allele if necessary). For the meQTL–CpG pairs, their effect sizes were measured by normalized regression coefficient $\widehat{\beta}=\beta / std\left(\beta \right)$, where *β* is the estimated regression coefficient and *std*(*β*) indicates the standard deviation of coefficient from meQTL analyses. The $\widehat{\beta}$ value represents the standardized methylation change related to an increase of one coding allele. Due to the rank normalization applied to the methylation values of blood, values of $\widehat{\beta}$ are not comparable across tissues, but the signs of $\widehat{\beta}$ reflect up- or down-regulation of SNPs on methylation, and the overall pattern of $\widehat{\beta}$ across the genome shows the relative strength of individual meQTLs. Thus, we computed the percentages of meQTL–CpG pairs showing the same or opposite signs of effects among tissues, and the Spearman’s rank correlations of the effects to evaluate the similarity of meQTL effect patterns among tissues.

### Enrichment test for meQTLs and targeted CpGs

To test the enrichment of meQTLs in previously published GWAS risk loci of various diseases (e.g, the NHGRI-EBI GWAS Catalog and psychiatric disorders) compared to non-meQTLs, we firstly pruned the whole SNP set with linkage disequilibrium (LD) r^2^ > 0.7 using the PLINK software. The LD pruning was supervised by GWAS risk loci so that risk SNPs were kept with high priorities. After the pruning process, the proportion of pruned meQTLs showing GWAS risk was calculated. We then generated a null distribution by randomly sampling 10^5^ sets of SNPs from the whole pruned SNP set. Each randomly picked SNP set had the same number of SNPs and similar MAF distribution as the pruned meQTLs. To ensure similar MAF distribution, we binned pruned meQTLs by MAF with intervals of 0.05, and then sampled the same number of SNPs with similar MAF distribution for each bin. For each random SNP set, the proportion of SNPs as GWAS risk loci was calculated. The percentage of sampled SNP sets having a higher proportion than the observed proportion was taken as the empirical *p* value, denoted by P_perm. The method was also used to test disease risk loci enrichment between cross-tissue meQTLs and combined meQTLs, and between combined meQTLs and non-meQTLs.

The same strategy was applied to the enrichment test for cross-tissue meQTLs in *cis*-eQTLs for brain and blood. The eQTLs in brain (frontal cortex Broadmann area 6) and blood (whole blood) were downloaded from GTEx (https://gtexportal.org/home/; version v6p). Only significant *cis*-eQTLs (within 1 Mbp, FDR < 0.05) were used for the enrichment test.

We evaluated the distribution of cross-tissue targeted CpGs in regions of gene body, TSS200, TSS1500, 3′ UTR, 5′ UTR, first exon, and enhancer, as well as regions (in terms of CpG density) of  CpG islands (CGIs), CGI shores, and CGI shelfs. Information on CpG distribution in these regions was from the published manifest file (https://support.illumina.com/downloads.html/). Enrichment tests in various regions were performed by two-sided Fisher’s 2 × 2 table exact tests which, for example, compared the odds of the CpGs being in the gene body when they were targeted by meQTLs in at least one tissue to the odds of the CpGs being in the gene body when they were not targeted by any meQTLs. Three types of comparisons were done: cross-tissue targeted CpGs vs tissue-specific meQTL-targeted CpGs; combined meQTL-targeted CpGs (CpGs targeted by meQTLs in at least one tissue) vs non-targeted CpGs (CpGs not targeted by meQTLs in any tissues); and cross-tissue meQTL-targeted CpGs vs non-targeted CpGs.

### Overlap of cross-tissue targeted CpGs with brain–blood correlated CpGs

To further characterize the extent of *cis*-meQTL effects on methylation variation across tissue types, we assessed the overlap between the CpGs targeted by cross-tissue meQTLs and the CpGs showing high correlations of methylation levels between brain (frontal cortex) and blood. From a published study [25], two levels of brain–blood correlation (r^2^ ≥ 25 and ≥ 50 %) were used to select 28,561 and 16,699 CpGs, respectively, which were filtered by aforementioned criteria and then used for Fisher’s exact enrichment test.

### Consensus co-methylation network analysis of cross-tissue targeted CpGs

A co-methylation network analysis was applied to cross-tissue meQTL-targeted CpGs to identify consensus networks across tissues using an R package for weighted correlation network analysis (WGCNA) [44]. Methylation data from brain (GSE74193; prefrontal cortex, 108 SZ patients and 136 controls), blood (GSE80417; 353 SZ patients and 322 controls), and saliva (described before) were obtained from three projects with both SZ patients and controls. The details of WGCNA can be seen in [44]. In brief, for each dataset the CpG adjacency matrix was calculated by a power of 6 of the correlation matrix among nodes (i.e., CpG), from which a topology overlap matrix (TOM) was derived to measure connection similarity among nodes (i.e., the overlap between any two nodes in terms of the extent they were connected to the same other nodes in the network). A consensus TOM across datasets was derived by defining the similarity of two nodes as the minimum similarity value among the datasets. Through the consensus TOM, an unsigned co-methylation network was constructed and densely interconnected CpGs were clustered into modules. Module eigengenes (ME), the first PC of methylation values of CpGs in a module, were computed for each tissue and tested for association with SZ diagnosis, controlling for the same covariates as used in the meQTL analysis. Association *p* values of ME in different tissues were then combined by Fisher’s combined method. Within a module, each CpG’s correlation with ME was computed for each tissue and the corresponding Z-scores across tissues were combined as the measure of each CpG’s module membership (MM) [45], indicating how close a CpG relates to the module. Each CpG’s association with SZ diagnosis was also computed and combined (Z-scores) across tissues, indicating its methylation significance (MS), from which the correlation between MM and MS for each CpG in the module was tested.

## Results

### meQTLs and targeted CpGs among tissues

**Fig. 1:**
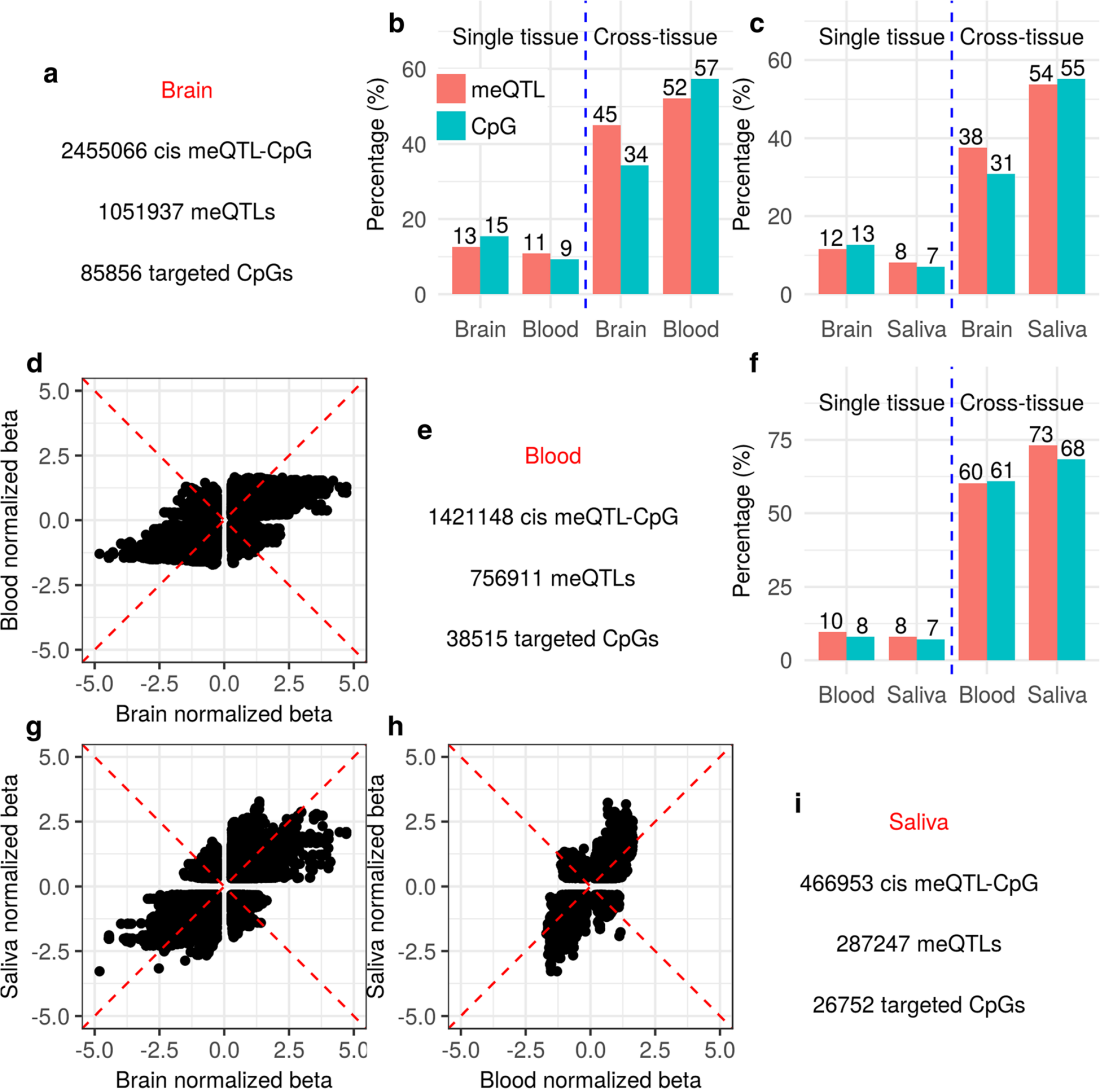
meQTLs and targeted CpGs among brain, blood, and saliva. **a**, **e**, **i** The numbers of meQTL–CpG pairs, involved meQTLs, and targeted CpGs from each tissue. **b**, **c**, **f** The percentages of meQTLs/targeted CpGs in each tissue (denoted by *Single tissue*) and percentages of cross-tissue meQTLs/targeted CpGs (denoted by *Cross-tissue*) in pair-wise tissue comparisons. *Red bars* indicate percentages of meQTLs and *blue bars* indicate percentages of targeted CpGs. **d**, **g**, **h** The $\widehat{\beta}$ values of matched meQTL–CpG pairs between tissues

The total numbers of SNPs, CpGs, *cis*-meQTL–CpG pairs, meQTLs, and targeted CpGs in each tissue and their overlap across tissues are provided in Additional file 1: Table S1. Figure 1a, e, i show the numbers of *cis* meQTL–CpG pairs, involved meQTLs, and targeted CpGs from each tissue. We conducted pair-wise tissue comparison as shown in Fig. 1b, c, f and 1d, g, h for brain vs blood, brain vs saliva, and blood vs saliva, respectively. Specific to each tested pair, common SNPs and CpGs were selected. In Fig. 1b–f, the percentages of common SNPs and CpGs having *cis*-meQTL effects are shown for each “single tissue”, while “cross-tissue” indicates the ratios of cross-tissue meQTLs or targeted CpGs over the total meQTLs or targeted CpGs in each tissue. When comparing brain with blood, 12.61 % of SNPs had *cis*-meQTL effects on 15.47 % of CpGs in brain, while 10.88 % of SNPs and 9.26 % of CpGs were detected with *cis* effects in blood. In both tissue types 528,286 meQTL–CpG pairs were shared, involving 45.04 % of brain meQTLs and 52.21 % of blood meQTLs, and 34.31 % of brain targeted CpGs and 57.28 % of blood targeted CpGs. These results are shown in Fig. 1b. When comparing brain with saliva as shown in Fig. 1c, 11.63 % of SNPs and 12.69 % of CpGs had *cis* effects in brain while 8.12 % of SNPs and 7.1 % of CpGs in saliva did. The 212,435 shared meQTL–CpG pairs involved 37.59 % meQTLs in brain and 53.83 % in saliva, and 30.8 % of targeted CpGs in brain and 55.12 % in saliva. When comparing blood with saliva as shown in Fig. 1f, 9.65 % of SNPs and 8.07 % of CpGs in blood, and 7.95 % of SNPs and 7.19 % of CpGs in saliva had *cis* effects. The 319,598 shared meQTL–CpG pairs involved 60.27 and 73.13 % of meQTLs and 60.96 and 68.36 % of targeted CpGs in blood and saliva, respectively.

Figure 1d–h show the *cis*-effect sizes ($\widehat{\beta}$ value) of the matched meQTL–CpG pairs between each pair of tissues: 84.8 % of the meQTL–CpG pairs have the same effect signs between brain and blood, 87.1 % between brain and saliva, and 92.9 % between blood and saliva, showing that majority of meQTLs have consistent effect signs across tissues. The rank correlations of effect sizes for the matched meQTL–CpG pairs were 0.78, 0.79, and 0.87 for brain vs blood, brain vs saliva, and blood vs saliva, respectively. By focusing on the meQTL–CpG pairs across all three tissues, we found similar correlations of 0.79, 0.79, and 0.88. Power analysis was performed to evaluate the meQTL detection power regarding sample size, effect size, and MAF, showing more power for meQTL detection in the blood study than the brain and saliva studies, especially when an meQTL has a smaller MAF or effect size, as shown in Additional file 1: Figure S1. When we restricted the analyses only to the meQTLs with consistent MAF across tissues (MAF difference < 0.1) and the effect sizes to have power over 0.8 for 200 samples (the smallest one of the three datasets), we observed similar ranges of meQTL overlap ratios (Additional file 2: Figure S2), indicating a relatively stable cross-tissue overlap.

Across all three tissues, we found 3,258,095 SNPs and 363,352 CpGs in common, resulting in 694,709, 564,150, and 430,956 *cis*-meQTL–CpG pairs in brain, blood, and saliva, respectively; 167,013 pairs were shared by all the tissues with 116,005 meQTLs and 10,879 targeted CpGs. The following analyses were conducted on these three-way cross-tissue meQTLs and targeted CpGs.

### Genomic distribution and functional annotation of cross-tissue targeted CpGs

**Fig. 2:**
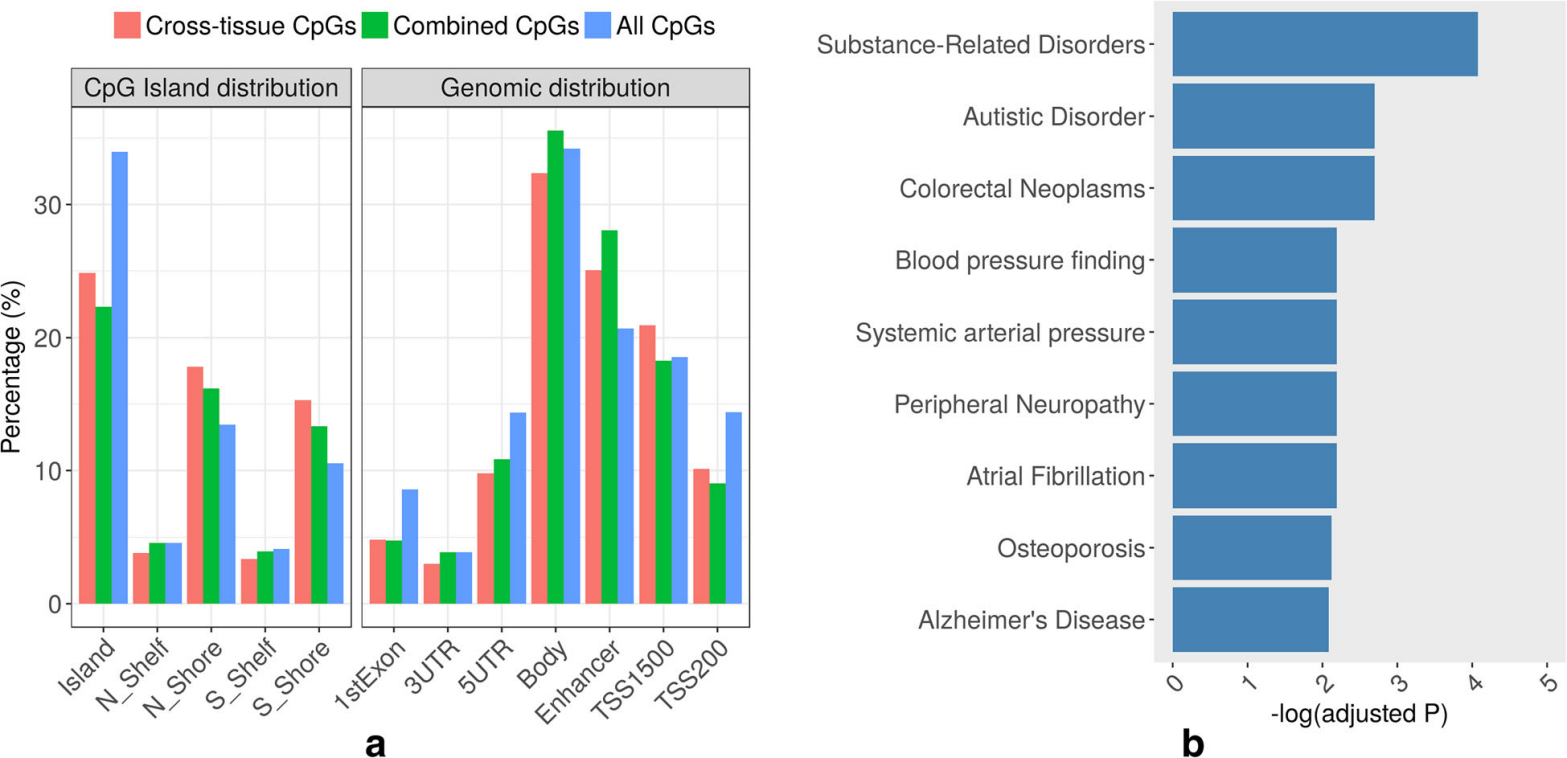
Characteristics of CpGs targeted by meQTLs. **a** The genomic and island distribution of CpGs targeted by meQTLs across three tissues (cross-tissue CpGs), CpGs targeted by meQTLs in at least one tissue (combined CpGs; consisting of cross-tissue and tissue-specific meQTL-targeted CpGs), and CpGs measured in all three tissues (all CpGs; consisting of combined CpGs and non-meQTL-targeted CpGs). **b** Enrichment of functional annotation of genes from cross-tissue targeted CpGs in complex diseases

Focusing on the cross-tissue meQTL-targeted CpGs (10,879), we explored their genomic distribution and compared them to those CpGs targeted by meQTLs in at least one tissue (combined CpGs; consisting of cross-tissue and tissue-specific meQTL-targeted CpGs) and total available CpGs (consisting of combined CpGs and non-meQTL-targeted CpGs). The percentage of CpGs located in the first exon, 3′ UTR, 5′ UTR, gene body, enhancer, TSS1500, and TSS200 regions are shown in Fig. 2a. Compared to non-targeted CpG sites, the combined CpGs were located more in enhancer regions (odds ratio (OR) = 1.64, *p* < 1 × 10^−200^) and gene body regions (OR = 1.07, *p* = 8.03 × 10^−5^) and depleted in the first exon (OR = 0.48, *p* < 1 × 10^−200^), 5′ UTR (OR = 0.69, *p* = 2 × 10^−172^), and TSS200 (OR = 0.54, *p* < 1 × 10^−200^) regions. Similarly, cross-tissue targeted CpGs were less distributed in the first exon (OR = 0.49, *p* = 2.87 × 10^−69^), 5′ UTR (OR = 0.49, *p* = 2.87 × 10^−69^), and TSS200 regions (OR = 0.49, *p* = 2.87 × 10^−69^) and more in enhancer regions (OR = 1.41, *p* = 1.32 × 10^−48^) and TSS1500 regions (OR = 1.16, *p* = 1.33 × 10^−9^). Compared to tissue-specific targeted CpGs (CpGs affected by meQTLs but not in all three tissue types), there were significantly higher proportions of cross-tissue targeted CpGs in TSS200 (OR = 1.16, *p* = 3.15 × 10^−5^) and TSS1500 regions (OR = 1.23, *p* = 6.13 × 10^−15^) but lower proportions in gene body regions (OR = 0.84, *p* = 1.08 × 10^−14^).

We also evaluated the location of the three sets of CpGs relative to CGIs. As shown in Fig. 2a, a significantly larger fraction of combined CpGs were located in CGI north shore (OR = 1.3, *p* = 2.8 × 10^−99^) and south shore (OR = 1.39, *p* = 9.73 × 10^−127^) regions compared to non-targeted CpGs. Interestingly, cross-tissue targeted CpGs had even higher enrichment in these two regions than tissue-specific targeted CpGs (OR = 1.15 and 1.21, *p* = 4.33 × 10^−7^ and 5.79 × 10^−11^, respectively). Combined CpGs and cross-tissue targeted CpGs were both less distributed in CGIs (OR = 0.5, *p* < 1 × 10^−200^; OR = 0.57, *p* = 3.19 × 10^−140^) compared to non-targeted CpGs.

We further tested enrichment of cross-tissue targeted CpGs in complex diseases (http://www.disgenet.org/web/DisGeNET/menu/home) and KEGG pathways by using the web tool Webgestalt. As shown in Fig. 2b, annotated genes from cross-tissue targeted CpGs were enriched in some psychiatric and neurological disorders such as substance-related disorders, autistic disorder, peripheral neuropathy, and Alzheimer’s disease (FDR < 0.01). In addition, Additional file 1: Table S2 lists the top ten involved pathways, of which some are related to neurodevelopment, even though their enrichment significance is marginal.

### Enrichment of cross-tissue meQTLs in complex diseases

**Fig. 3:**
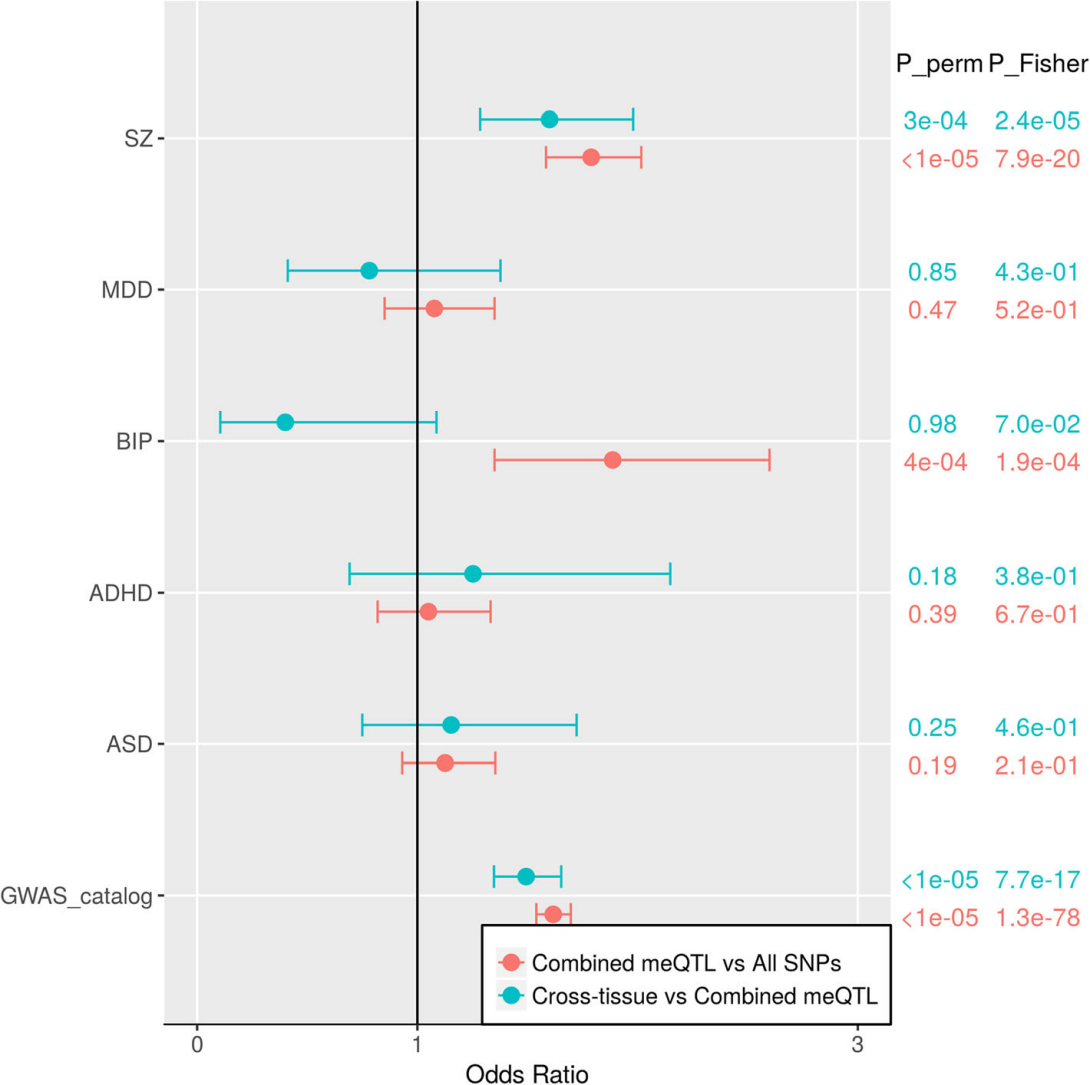
Enrichment tests for cross-tissue meQTLs and combined meQTLs in the risk loci for five psychiatric disorders from Psychiatric Genomics Consortium reports and 996 traits/diseases from the NHGRI-EBI GWAS Catalog. *SZ* schizophrenia, *MDD* major depression disorder, *BIP* bipolar disorders, *ADHD* attention deficit hyperactivity disorder, *ASD* autism disorder. The odds ratio, 95 % confidence interval, and two *p* values from permutation (P_perm) and Fisher’s exact test (P_Fisher) are listed for each enrichment test

Some studies have shown a mediation effect of DNA methylation on the genetic risk for complex diseases [46], especially psychiatric disorders [14]. We evaluated the enrichment in various diseases of SNPs showing *cis*-meQTL effects across all three tissues (cross-tissue meQTLs) and SNPs showing meQTL effects in at least one tissue (combined meQTLs; consisting of cross-tissue and tissue-specific meQTLs), as shown in Fig. 3. First we tested the enrichment in reported GWAS risk loci of diverse diseases from the NHGRI-EBI GWAS Catalog (database download 2017-3-6). There were 26,625 genome-wide significant risk loci from 1764 disease traits, and 12,451 SNPs involved in 966 diseases/traits were included in our study by matching rs numbers. Of the GWAS risk SNPs, 2956 were meQTLs in at least one of three tissues (23.74 % of GWAS risk SNPs, 0.56 % of combined meQTLs), showing significant enrichment (OR = 1.62, permutation P_perm < 1 × 10^−5^, Fisher’s exact test P_Fisher = 1.3 × 10^−78^) compared to non-meQTL SNPs. And 706 GWAS risk SNPs were cross-tissue meQTLs (23.89 % of GWAS risk meQTLs, 0.61 % of cross-tissue meQTLs). Cross-tissue meQTLs were even enriched in GWAS risk SNPs compared to tissue-specific meQTLs (OR = 1.49, P_perm < 1 × 10^−5^, P_Fisher = 5 × 10^−14^).

Focusing on psychiatric disorders, we tested the enrichment of different sets of meQTLs in the GWAS risk loci of the five disorders: SZ, major depression disorder (MDD), BIP, attention deficit hyperactivity disorder (ADHD), and ASD. The GWAS risk loci were obtained from the mega analyses reported by the Psychiatric Genomics Consortium. We used *p* < 1 × 10^−5^ for SZ and *p* < 1 × 10^−3^ for other psychiatric disorders to select risk loci from these reports. When compared to non-meQTLs, combined meQTLs showed significant enrichment for genetic risk loci of BIP and SZ. When comparing cross-tissue meQTLs with tissue-specific meQTLs, cross-tissue meQTLs were again significantly enriched for SZ risk loci, but not for BIP. Noticeably, both combined meQTLs and cross-tissue meQTLs had higher proportions of SZ risk loci than non-meQTLs (OR = 1.79 and 2.49, respectively). In detail, among 18,761 SZ risk loci in our data, 4452 SNPs (23.73 %) were meQTLs in at least one tissue and 1496 (7.97 %) were cross-tissue meQTLs. After applying a more strict threshold (*p* < 1 × 10^−7^) for SZ risk, which resulted in 7936 SZ risk loci, we found 2299 (28.97 %) were combined meQTLs with OR = 1.86 (P_perm < 1 × 10^−5^ and P_Fisher = 9 × 10^−10^). Of these SZ risk combined meQTLs, 33.5 % were cross-tissue meQTLs (OR = 1.72, P_perm < 9 × 10^−4^ and P_Fisher = 1.3 × 10^−3^), including five genome-wide significant index SNPs [47]. The CpGs targeted by cross-tissue meQTLs with SZ risk were mainly mapped to genes *BTN3A2, HLA-DQA1, MAD1L1, ZNF389, PPP1R13B, TSNARE1, HLA-C, SMG6, SRR, AS3MT, LOC285830, ITIH4, and MUSTN1* (listed in Additional file 2).

### Overlap between meQTLs and eQTLs across tissue types

DNA methylation has been considered as a regulator of gene expression, especially when located close to the transcription start site of genes. To evaluate the genetic influence on both DNA methylation and gene expression, we tested the overlap of *cis*-meQTLs with *cis*-eQTLs in brain and blood. *cis*-eQTLs (SNP gene distance < 1 Mbps) from brain (frontal cortex Broadmann area 9) and whole blood were downloaded from GTEx project (V6p release). We included 139,747 brain eQTLs (FDR < 5 %) in our brain SNP data, and 588,981 blood eQTLs in our blood SNP data, where 45.5 % of brain eQTLs (63,579) and 28.68 % of blood eQTLs (168,941) were also meQTLs in each tissue, respectively. We further tested the enrichment of cross-tissue meQTLs in cross-tissue eQTLs. There were 39,653 eQTLs targeting the same gene in both tissues and 7372 eQTLs (18.59 %) were also meQTLs (6.35 %) across tissues, showing significant enrichment of cross-tissue meQTLs in cross-tissue eQTLs (OR = 8.75, P_perm < 1 × 10^−5^, P_Fisher < 1 × 10^−200^) compared to non-meQTLs. Interestingly, among the overlapping cross-tissue eQTLs and meQTLs, 351 QTLs were mainly located in chromosome 6p21.1–6p24.3 regions (Additional file 3) and showed significant SZ risk with *P* < 1 × 10^−5^, strongly suggesting a biological pathway from these SZ genetic risk factors to the disease through regulating methylation and gene expression.

### Correlation of cross-tissue targeted CpG methylation in brain and blood

The meQTL effect has been suggested to contribute to the correlation of DNA methylation across tissues [25]. We compared cross-tissue targeted CpGs and CpGs whose methylation values were highly correlated between brain and blood. From the study by Hannon et al. [25], two levels of correlation between brain (frontal cortex) and blood were used in our analyses with r^2^ > 25 and > 50 %, resulting in 15,207 and 7479 CpGs, respectively. Of the CpGs, 21 and 15.62 % in each set were targeted by cross-tissue meQTLs. Enrichment tests showed that CpGs targeted by cross-tissue meQTLs more likely had high cross-tissue correlations (OR(r^2^ > 25 %) = 11.78, P_Fisher < 1 × 10^−200^; OR(r^2^ > 50 %) = 6.6, P_Fisher < 1 × 10^−200^) compared to the others. Along with the increase of the meQTL effect, there was an increase of probability of targeted CpGs showing high cross-tissue correlation of methylation levels, as shown in Additional file 1: Figure S3.

### Consensus co-methylation networks across tissues and their relationship to SZ

Since cross-tissue meQTLs have shown significant enrichment in SZ risk loci, it is valuable to test directly if their targeted CpGs demonstrate a relationship with SZ across tissues. By focusing on cross-tissue targeted CpGs, we conducted a consensus WGCNA across tissues. Instead of single CpG sites, we attempted to identify the cross-tissue CpG modules related to SZ. One consensus module was identified across the three tissue types but only showed marginal association with SZ in blood (*P* = 0.08) after controlling for covariates (age, sex, batch, cell type, and smoking). By constructing the network in a pair-wise fashion, we found two brain–blood consensus modules, with one showing significant negative SZ associations in both tissues (P_brain = 5.33 × 10^−3^, T = −2.81; P_blood = 2.87 × 10^−4^, T = −3.65; combined *p* = 5.5 × 10^−6^), while no SZ-related modules were identified in brain and saliva or blood and saliva pairs. There were 962 CpGs included in the consensus SZ-related module between brain and blood, with module membership of each CpG closely correlated with the combined SZ-relevance Z-score (r = −0.53).

## Discussion

We present a comprehensive analysis of *cis*-meQTLs across brain, blood, and saliva. Large proportions of meQTLs (38–73 %) and targeted CpGs (31–68 %) were shared among tissues, which are higher than in previous reports based on the HumanMethylation27 (HM27k) array (6.6–35 %) [10]. This may be due to a larger sample size and higher resolution of SNP and methylation arrays (HM450k) in this study. While the HM27k array mainly profiled CpGs in promoter regions, we found the targeted CpGs were enriched in gene bodies, enhancer regions, and further away at transcription start sites (TSSs) such as TSS1500. This finding was in line with previous studies in multiple cell lines showing that meQTLs more likely reside at distant regulatory elements than at promoters [12, 30]. In addition, we found enrichment of meQTL-targeted CpGs in CGI shore regions, consistent with other meQTL analyses [16, 30]. Cross-tissue targeted CpGs showed even higher proportions in CGI shore regions than tissue-specific targeted CpGs. This observation complements previous reports on CpGs in CGI shores showing more variation and more involvement in various diseases [48].

For the shared meQTLs among tissues, we found overall high similarities of meQTL effects in terms of effect sign (85–93 % consistent) and pattern (correlation = 0.78–0.87), with a slightly higher similarity between blood and saliva. No marked differences between similarities of blood and saliva to the brain were seen. On the other hand, there were also a large number of tissue-specific meQTLs—around 27–69 % of meQTLs were tissue-specific, which was also reported in previous studies [10, 18]. The large percentage of tissue-specific meQTLs may be due, in part, to differences between the three studies, including different meQTL detection power because of varying sample sizes in tissues, minor allele frequency differences among cohorts, as shown in our power analysis and another report [10], slightly different analytic approaches (e.g., rank normalization in blood methylation), and other unmeasured confounding factors. Although we have also found consistent meQTL overlap ratios among tissues when applying more conservative criteria, as shown in Additional file 1: Fig. S2, the effect of cohort-related differences cannot be totally ruled out. Nevertheless, our study focuses on the identified cross-tissue meQTLs given each study performed reasonable false positive control.

We found a large overlap between *cis*-meQTLs and *cis*-eQTLs in both brain (46 %) and blood (29 %), which is much higher than the previously reported 5 % of QTLs associated with both proximal DNA methylation and gene expression [15, 16]. The previous studies were based on the HM27k methylation array, which results in promoter-biased profiling as mentioned above. For the shared meQTL–eQTLs in our findings, the median distance between target gene and targeted CpG was 27.4 kbp with an interquartile range of 4–75.8 kbp, showing that a majority of CpGs (80.3 % with distance to TSS > 1500 bp) were located outside promoters. This significant overlap was also in line with a recent study of fetal meQTLs which reported significant enrichment of fetal *cis*-meQTLs in *cis*-eQTLs, suggesting a high probability of both QTLs being located farther away from the gene TSS [5]. We further found significant enrichment of cross-tissue *cis*-meQTLs in cross-tissue *cis*-eQTLs. Despite the lack of gene expression and DNA methylation data to validate a pathological mechanism, some cross-tissue *cis*-meQTLs have been previously reported to regulate gene expression through nearby methylation [15, 16], suggesting a potential role of methylation in mediating the effects of these QTLs (both meQTLs and eQTLs) on gene expression.

meQTLs were broadly enriched in risk loci of common diseases and traits and some psychiatric disorders (i.e., BIP and SZ, though cross-tissue meQTLs were not enriched for BIP, maybe indicating tissue specificity of BIP pathology). Especially for SZ, in line with previous studies in brain and blood [5, 49], we found significantly higher proportions of meQTLs and cross-tissue meQTLs as SZ risk loci, indicating the complex genetic mechanism of SZ and the possible role of surrogate peripheral tissues in studying the pathology of SZ via these meQTLs. Furthermore, by matching meQTLs, eQTLs, and SZ risk loci, we identified a set of SNPs. Some influence both nearby CpG methylation and expression of genes, including *BTN3A2*, *ITIH4*, *HCG27*, and *HLA-C*. Their targeted CpGs were located within 820 bps of the target gene TSS. Other SNPs nearby genes *ZKSCAN8, HMOX2, C2orf69, CYP2D6, NT5DC2, C10orf32-ASMT, HLA-C,HLA-G, HLA-DRB5,* and *HLA-DQB1* regulate both methylation and gene expression from further distances (median distance = 73.5 kbp, interquartile range = 37–98.2 kbp, data not shown), suggesting possible regulation of methylation from distal gene regulatory regions, which is consistent with our finding on genomic distribution of cross-tissue targeted CpGs.

We found that CpGs targeted by meQTLs across tissues were more likely to show methylation correlation among tissues compared to the other CpGs. CpGs with higher meQTL effects were more likely correlated across tissues. A further *t*-test showed significant differences of meQTL effects between cross-tissue targeted CpGs and tissue-specific targeted CpGs (*t* = 64.181, *p* value < 1 × 10^−200^), suggesting a potential contribution of the genetic component to the cross-tissue targeted CpG correlation.

Consensus co-methylation network analysis identified one module common to the three tissues, although not significantly related to SZ, but provided evidence of cross-tissue CpG correlation. One consensus co-methylation module in brain and blood was identified to be associated with SZ but not replicated in saliva, suggesting a closer relationship of the co-methylation network between brain and blood with SZ. Note that none of the cross-tissue targeted CpGs showed differences between SZ patients and controls, passing multiple comparison correction as reported in a previous work on brain methylation [1]. We found that some CpGs with moderate group differences were highly correlated to form a network that showed a significant association with SZ in our analyses. Our finding suggests that more powerful multivariate statistical models are needed for differential methylation analyses in order to account for co-methylation structures.

The findings of this study should be interpreted with regard to several limitations. Only *cis*-acting SNP–CpG effects were investigated. Previous studies have reported *trans*-meQTLs at very small percentages (2–7 % meQTLs are *trans*) but that are highly polygenic [5, 13]. Such *trans*-meQTL effects as well as a potential regulatory mechanism due to the 3D chromatin structure [50] will be interesting to study in future analyses. Secondly, data sets were collected from different projects with different population backgrounds, sample sizes, and potentially many other covariates. Although top ancestry-related PCs and covariates (e.g., cell type, age, sex) were used to adjust the meQTL analysis, we cannot rule out the influence of other confounding factors. In addition, we focused our analysis only on overlapping SNPs across studies, which may limit our meQTL detection. Thirdly, saliva data were assayed by Illumina Methylation EPIC array. Although the EPIC array can cover almost 93 % of HM450k array probes used for brain and blood, some probes were still not captured in the cross-tissue analysis. Two different Illumina arrays were used for saliva sample genotyping. Although imputation was applied to genotyped data separately using the same protocol, and only loci with high imputation quality were kept and merged, we cannot ensure the removal of batch effects from the results. In addition, the saliva data included both cases and controls. Although group information has been added as a covariate in the saliva meQTL analysis, it may reduce the meQTL effects when SNPs or CpGs are highly associated with the group variable. Finally, due to limited access to the original data from brain and blood tissues, we set a unified conservative threshold of *p* < 1 × 10^−5^ to the meQTL significance instead of a FDR-corrected threshold. And for blood meQTLs, we only had access to partial meQTLs whose *p* values are between 1 × 10^−7^ and 1 × 10^−5^, but all meQTLs less than 1 × 10^−7^.

## Conclusions

We leveraged genotype and DNA methylation data from brain, blood, and saliva to systematically characterize *cis*-meQTLs and their targeted CpGs among tissues. We identified significant overlap of meQTLs and targeted CpGs across tissues, where cross-tissue targeted CpGs are proportionally located more in enhancer regions and tend to show high methylation correlation among tissues. A large portion of meQTLs also had a tissue-specific effect especially in brain, showing the potential function of these meQTLs in influencing brain methylation or gene expression. Compared to tissue-specific meQTLs and non-meQTLs, cross-tissue meQTLs were more enriched for eQTLs than previously observed, and more likely to be risk loci for SZ. With similar co-methylation networks identified across tissues, our findings suggest the potential of cross-tissue meQTLs for studying the genetic effect on SZ. The study provides compelling motivation for a well-designed experiment to further validate the use of surrogate tissues in the study of psychiatric disorders.

## Additional files

Additional file 1: Supplementary file including power analysis plot for meQTL detection (Figure S1), meQTL overlap among tissues by restricting SNPs and effect sizes (Figure S2), plot of the relationship between meQTL effect and the proportion of highly correlated CpGs in brain and blood (Figure S3), numbers of SNPs, CpGs, *cis*-SNP–CpG pairs, meQTLs, and targeted CpGs in each tissue and their overlap across tissues (Additional file 1: Table S1), and the top ten pathways involved according to genes annotated from cross-tissue targeted CpGs (Additional file 1: Table S2). (DOCX 606 kb)

Additional file 2: Supplementary data file listing the SNPs which are cross-tissue meQTLs and SZ risks (*p* < 10^−5^) reported by the PGC SZ study. (CSV 163 kb)

Additional file 3: Supplementary data file listing the SNPs which are cross-tissue meQTLs, cross-tissue eQTLs, and SZ risks (*p* < 10^−5^). (CSV 153 kb)

## Abbreviations

ADHD: Attention deficit hyperactivity disorder
ASD: Autism spectrum disorder
BIP: Bipolar disorder
CGI: CpG island
eQTL: Expression quantitative trait loci
HM27k: HumanMethylation27k
LD: Linkage disequilibrium
MAF: Minor allele frequency
MDD: Major depressive disorder
ME: Module eigengenes
meQTL: Methylation quantitative trait loci
MM: Module membership
MS: Methylation significance
PC: Principle component
SNP: Single nucleotide polymorphism
SZ: Schizophrenia
TOM: Topology overlap matrix
TSS: Transcription start site
WGCNA: Weighted correlation network analysis

## References

[1] Jaffe AE, Gao Y, Deep-Soboslay A, Tao R, Hyde TM, Weinberger DR, Kleinman JE (2016). Mapping DNA methylation across development, genotype and schizophrenia in the human frontal cortex. Nat Neurosci..

[2] Vogel Ciernia A, LaSalle J (2016). The landscape of DNA methylation amid a perfect storm of autism aetiologies. Nat Rev Neurosci..

[3] Grayson DR, Guidotti A (2013). The dynamics of DNA methylation in schizophrenia and related psychiatric disorders. Neuropsychopharmacology..

[4] Pidsley R, Viana J, Hannon E, Spiers H, Troakes C, Al-Saraj S, Mechawar N, Turecki G, Schalkwyk LC, Bray NJ (2014). Methylomic profiling of human brain tissue supports a neurodevelopmental origin for schizophrenia. Genome Biol..

[5] Hannon E, Spiers H, Viana J, Pidsley R, Burrage J, Murphy TM, Troakes C, Turecki G, O'Donovan MC, Schalkwyk LC (2016). Methylation QTL in the developing brain and their enrichment in schizophrenia risk loci. Nat Neurosci..

[6] Teroganova N, Girshkin L, Suter CM, Green MJ (2016). DNA methylation in peripheral tissue of schizophrenia and bipolar disorder: a systematic review. BMC Genet..

[7] Liu J, Chen J, Ehrlich S, Walton E, White T, Perrone-Bizzozero N, Bustillo J, Turner JA, Calhoun VD (2014). Methylation patterns in whole blood correlate with symptoms in schizophrenia patients. Schizophr Bull..

[8] Melas PA, Rogdaki M, Osby U, Schalling M, Lavebratt C, Ekstrom TJ (2012). Epigenetic aberrations in leukocytes of patients with schizophrenia: association of global DNA methylation with antipsychotic drug treatment and disease onset. FASEB J..

[9] Walton E, Liu J, Hass J, White T, Scholz M, Roessner V, Gollub R, Calhoun VD, Ehrlich S (2014). MB-COMT promoter DNA methylation is associated with working-memory processing in schizophrenia patients and healthy controls. Epigenetics..

[10] Smith AK, Kilaru V, Kocak M, Almli LM, Mercer KB, Ressler KJ, Tylavsky FA, Conneely KN (2014). Methylation quantitative trait loci (meQTLs) are consistently detected across ancestry, developmental stage, and tissue type. BMC Genomics..

[11] Bell JT, Pai AA, Pickrell JK, Gaffney DJ, Pique-Regi R, Degner JF, Gilad Y, Pritchard JK (2011). DNA methylation patterns associate with genetic and gene expression variation in HapMap cell lines. Genome Biol..

[12] Banovich NE, Lan X, McVicker G, van de Geijn B, Degner JF, Blischak JD, Roux J, Pritchard JK, Gilad Y (2014). Methylation QTL are associated with coordinated changes in transcription factor binding, histone modifications, and gene expression levels. PLoS Genet..

[13] Gaunt TR, Shihab HA, Hemani G, Min JL, Woodward G, Lyttleton O, Zheng J, Duggirala A, McArdle WL, Ho K (2016). Systematic identification of genetic influences on methylation across the human life course. Genome Biol..

[14] Lin D, Chen J, Ehrlich S, Bustillo JR, Perrone-Bizzozero N, Walton E, Clark VP, Wang YP, Sui J, Du Y, et al. Cross-Tissue Exploration of Genetic and Epigenetic Effects on Brain Gray Matter in Schizophrenia. Schizophr Bull. 2018;44(2):443–52. 10.1093/schbul/sbx068 PMC5814943 28521044

[15] Gibbs JR, van der Brug MP, Hernandez DG, Traynor BJ, Nalls MA, Lai SL, Arepalli S, Dillman A, Rafferty IP, Troncoso J (2010). Abundant quantitative trait loci exist for DNA methylation and gene expression in human brain. PLoS Genet..

[16] Zhang D, Cheng L, Badner JA, Chen C, Chen Q, Luo W, Craig DW, Redman M, Gershon ES, Liu C (2010). Genetic control of individual differences in gene-specific methylation in human brain. Am J Hum Genet..

[17] Maurano MT, Humbert R, Rynes E, Thurman RE, Haugen E, Wang H, Reynolds AP, Sandstrom R, Qu H, Brody J (2012). Systematic localization of common disease-associated variation in regulatory DNA. Science..

[18] Andrews SV, Ellis SE, Bakulski KM, Sheppard B, Croen LA, Hertz-Picciotto I, Newschaffer CJ, Feinberg AP, Arking DE, Ladd-Acosta C, et al. Cross-tissue integration of genetic and epigenetic data offers insight into autism spectrum disorder. bioRxiv. 2016; 10.1038/s41467-017-00868-y PMC5654961 29066808

[19] Gamazon E, Badner J, Cheng L, Zhang C, Zhang D, Cox N, Gershon E, Kelsoe J, Greenwood T, Nievergelt C (2013). Enrichment of cis-regulatory gene expression SNPs and methylation quantitative trait loci among bipolar disorder susceptibility variants. Mol Psychiatry..

[20] Numata S, Ye T, Herman M, Lipska BK (2014). DNA methylation changes in the postmortem dorsolateral prefrontal cortex of patients with schizophrenia. Front Genet..

[21] Bakulski KM, Halladay A, Hu VW, Mill J, Fallin MD (2016). Epigenetic research in neuropsychiatric disorders: the “tissue issue**”**. Curr Behav Neurosci Rep..

[22] Davies MN, Volta M, Pidsley R, Lunnon K, Dixit A, Lovestone S, Coarfa C, Harris RA, Milosavljevic A, Troakes C (2012). Functional annotation of the human brain methylome identifies tissue-specific epigenetic variation across brain and blood. Genome Biol..

[23] Horvath S, Zhang Y, Langfelder P, Kahn RS, Boks MP, van Eijk K, van den Berg LH, Ophoff RA (2012). Aging effects on DNA methylation modules in human brain and blood tissue. Genome Biol..

[24] Walton E, Hass J, Liu J, Roffman JL, Bernardoni F, Roessner V, Kirsch M, Schackert G, Calhoun V, Ehrlich S. Correspondence of DNA Methylation Between Blood and Brain Tissue and Its Application to Schizophrenia Research. Schizophr Bull. 2016;42(2):406–14. 10.1093/schbul/sbv074 PMC4753587 26056378

[25] Hannon E, Lunnon K, Schalkwyk L, Mill J (2015). Interindividual methylomic variation across blood, cortex, and cerebellum: implications for epigenetic studies of neurological and neuropsychiatric phenotypes. Epigenetics..

[26] Consortium GT (2015). The Genotype-Tissue Expression (GTEx) pilot analysis: multitissue gene regulation in humans. Science..

[27] Quon G, Lippert C, Heckerman D, Listgarten J (2013). Patterns of methylation heritability in a genome-wide analysis of four brain regions. Nucleic Acids Res..

[28] McRae AF, Powell JE, Henders AK, Bowdler L, Hemani G, Shah S, Painter JN, Martin NG, Visscher PM, Montgomery GW (2014). Contribution of genetic variation to transgenerational inheritance of DNA methylation. Genome Biol..

[29] Kaminsky ZA, Tang T, Wang SC, Ptak C, Oh GH, Wong AH, Feldcamp LA, Virtanen C, Halfvarson J, Tysk C (2009). DNA methylation profiles in monozygotic and dizygotic twins. Nat Genet..

[30] Gutierrez-Arcelus M, Ongen H, Lappalainen T, Montgomery SB, Buil A, Yurovsky A, Bryois J, Padioleau I, Romano L, Planchon A (2015). Tissue-specific effects of genetic and epigenetic variation on gene regulation and splicing. PLoS Genet..

[31] Aine CJ, Bockholt HJ, Bustillo JR, Canive JM, Caprihan A, Gasparovic C, Hanlon FM, Houck JM, Jung RE, Lauriello J (2017). Multimodal neuroimaging in schizophrenia: description and dissemination. Neuroinformatics..

[32] Bustillo JR, Jones T, Chen H, Lemke N, Abbott C, Qualls C, Stromberg S, Canive J, Gasparovic C. Glutamatergic and neuronal dysfunction in gray and white matter: a spectroscopic imaging study in a large schizophrenia sample. Schizophr Bull. 2016; 10.1093/schbul/sbw122 PMC5473520 27550776

[33] Howie B, Fuchsberger C, Stephens M, Marchini J, Abecasis GR (2012). Fast and accurate genotype imputation in genome-wide association studies through pre-phasing. Nat Genet..

[34] Aryee MJ, Jaffe AE, Corrada-Bravo H, Ladd-Acosta C, Feinberg AP, Hansen KD, Irizarry RA (2014). Minfi: a flexible and comprehensive Bioconductor package for the analysis of Infinium DNA methylation microarrays. Bioinformatics..

[35] Chen YA, Lemire M, Choufani S, Butcher DT, Grafodatskaya D, Zanke BW, Gallinger S, Hudson TJ, Weksberg R (2013). Discovery of cross-reactive probes and polymorphic CpGs in the Illumina Infinium HumanMethylation450 microarray. Epigenetics..

[36] Naeem H, Wong NC, Chatterton Z, Hong MK, Pedersen JS, Corcoran NM, Hovens CM, Macintyre G (2014). Reducing the risk of false discovery enabling identification of biologically significant genome-wide methylation status using the HumanMethylation450 array. BMC Genomics..

[37] Rahmani E, Yedidim R, Shenhav L, Schweiger R, Weissbrod O, Zaitlen N, Halperin E (2017). GLINT: a user-friendly toolset for the analysis of high-throughput DNA-methylation array data. Bioinformatics..

[38] Troyanskaya O, Cantor M, Sherlock G, Brown P, Hastie T, Tibshirani R, Botstein D, Altman RB (2001). Missing value estimation methods for DNA microarrays. Bioinformatics..

[39] Johnson WE, Li C, Rabinovic A (2007). Adjusting batch effects in microarray expression data using empirical Bayes methods. Biostatistics..

[40] Leek JT, Johnson WE, Parker HS, Jaffe AE, Storey JD (2012). The sva package for removing batch effects and other unwanted variation in high-throughput experiments. Bioinformatics..

[41] Houseman EA, Accomando WP, Koestler DC, Christensen BC, Marsit CJ, Nelson HH, Wiencke JK, Kelsey KT (2012). DNA methylation arrays as surrogate measures of cell mixture distribution. BMC Bioinformatics..

[42] Shabalin AA (2012). Matrix eQTL: ultra fast eQTL analysis via large matrix operations. Bioinformatics..

[43] Grundberg E, Meduri E, Sandling JK, Hedman ÅK, Keildson S, Buil A, Busche S, Yuan W, Nisbet J, Sekowska M (2013). Global analysis of DNA methylation variation in adipose tissue from twins reveals links to disease-associated variants in distal regulatory elements. Am J Hum Genet..

[44] Langfelder P, Horvath S (2008). WGCNA: an R package for weighted correlation network analysis. BMC Bioinformatics..

[45] Willer CJ, Li Y, Abecasis GR (2010). METAL: fast and efficient meta-analysis of genomewide association scans. Bioinformatics..

[46] Liu Y, Aryee MJ, Padyukov L, Fallin MD, Hesselberg E, Runarsson A, Reinius L, Acevedo N, Taub M, Ronninger M (2013). Epigenome-wide association data implicate DNA methylation as an intermediary of genetic risk in rheumatoid arthritis. Nat Biotechnol..

[47] Schizophrenia Working Group of the Psychiatric Genomics Consortium (2014). Biological insights from 108 schizophrenia-associated genetic loci. Nature..

[48] Irizarry RA, Ladd-Acosta C, Wen B, Wu Z, Montano C, Onyango P, Cui H, Gabo K, Rongione M, Webster M (2009). The human colon cancer methylome shows similar hypo-and hypermethylation at conserved tissue-specific CpG island shores. Nat Genet..

[49] McClay JL, Shabalin AA, Dozmorov MG, Adkins DE, Kumar G, Nerella S, Clark SL, Bergen SE, Hultman CM, Magnusson PK (2015). High density methylation QTL analysis in human blood via next-generation sequencing of the methylated genomic DNA fraction. Genome Biol..

[50] Won H, de la Torre-Ubieta L, Stein JL, Parikshak NN, Huang J, Opland CK, Gandal MJ, Sutton GJ, Hormozdiari F, Lu D (2016). Chromosome conformation elucidates regulatory relationships in developing human brain. Nature..

