# Supplementary material for: Characterization of cross-tissue genetic-epigenetic effects and their patterns in schizophrenia
Source: Genome Med. 2018 Feb 26;10:13. doi: 10.1186/s13073-018-0519-4 (PMC5828480; doi:10.1186/s13073-018-0519-4)
Supplement: Supplementary file 1 — Supplementary file including power analysis plot for meQTL detection (Figure S1), meQTL overlap among tissues by restricting SNPs and effect sizes (Figure S2), plot of the relationship between meQTL effect and the proportion of highly correlated CpGs in brain and blood (Figure S3), numbers of SNPs, CpGs, cis-SNP–CpG pairs, meQTLs, and targeted CpGs in each tissue and their overlap across tissues (Additional file 1: Table S1), and the top ten pathways involved according to genes annotated from cross-tissue targeted CpGs (Additional file 1: Table S2). (DOCX 606 kb) [file 13073_2018_519_MOESM1_ESM.docx]

# Supplementary Information:

## 1) MeQTL detection power analysis

## To evaluate the capability of detecting meQTL effects, we performed power analysis regarding the number of sample size (n = 100~1000), effect size (normalized regression coefficient $\hat{\boldsymbol{\beta}}$: 0.3 ~ 1.9, with a similar range as in real data ) and minor allele frequency (MAF = 0.05 to 0.4). We generated genotype data first by categorizing a random vector into three levels (0,1,2) based on the MAF [[1](#_ENREF_1)]. Coefficient vector $\boldsymbol{\beta}$ was then randomly selected from a normal distribution $\boldsymbol{\beta\sim N}\left( \hat{\boldsymbol{\beta}}\boldsymbol{,1} \right)$ and methylation values were simulated by $\boldsymbol{M=}\hat{\boldsymbol{\beta}}\boldsymbol{\times SNP}$. For each setting of parameters, we simulated 1000 times and counted the proportion of successful detection with type I error as p<0.05. The results are plotted in Fig S1. Furthermore, we calculated the percentage of detected coefficients with opposite direction to the given effect as the probability of detecting false opposite direction of meQTLs. The rate was less than 1% in all of combinations of parameters (i.e, effect size and MAF) with most of them were exactly 0, indicating rare possibility for wrongly detection of meQTL effect sign.

##
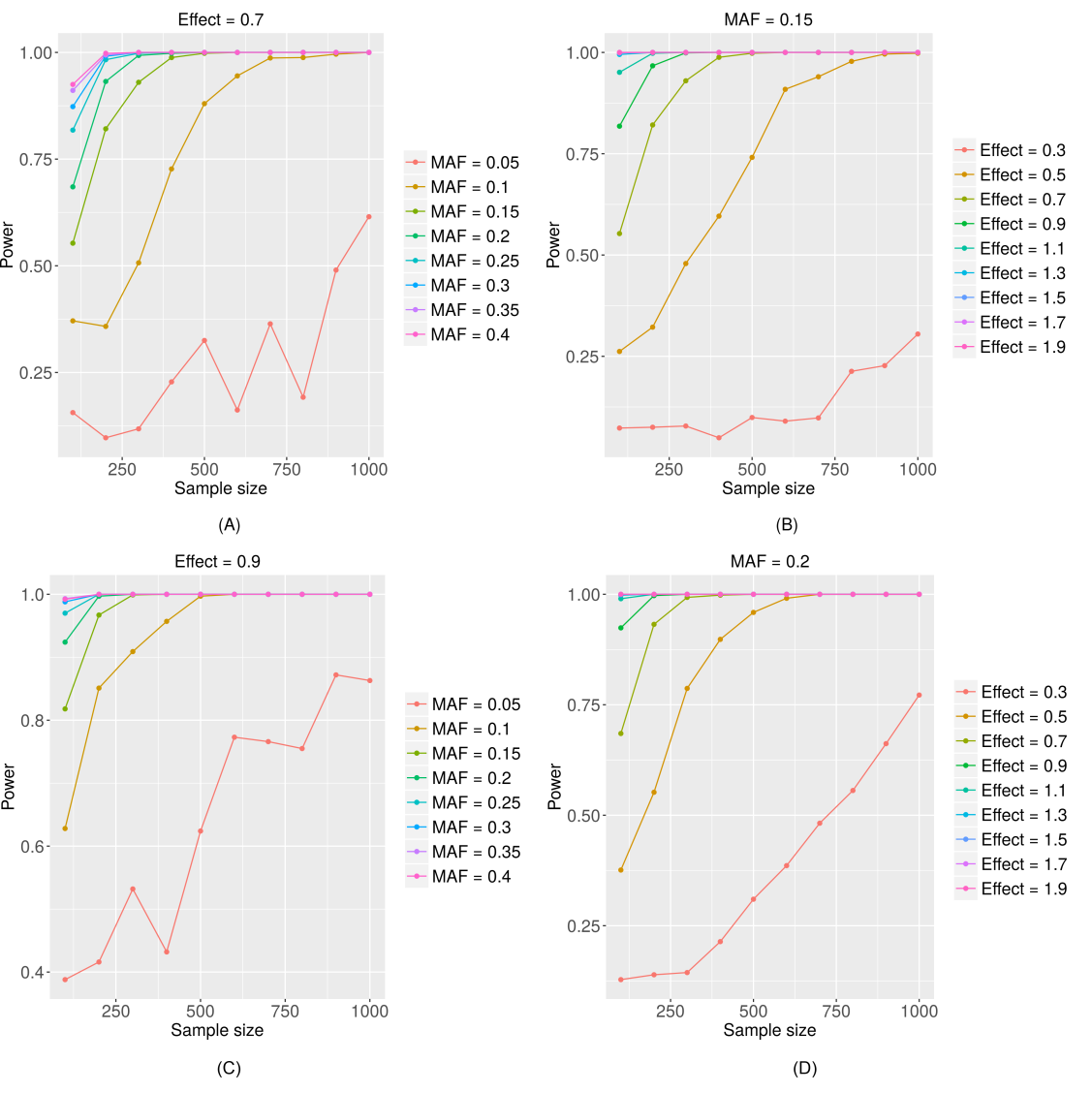


## Figure S1. Power analyses on meQTL detection. (A,C): the power when effect size $\hat{\boldsymbol{\beta}}$ =0.7 and 0.9 respectively, with sample size changing from 100 to 1000, and MAF changing from 0.05 to 0.4. (B,D): the power when MAF =0.15 and 0.2 respectively, with effect size $\hat{\boldsymbol{\beta}}$ changing from 0.3 to 1.9, and sample size changing from 100 to 1000.

**2) MeQTLs overlap among tissues on restricted SNPs and effect sizes**

Effect size and minor allele frequency (MAF) are the two main factors among others and can be modeled and tested for power analyses. Different sample sizes would influence the power to detect meQTLs with different effect sizes and MAFs. Each of the three studies analyzed here has its own power to detect meQTLs; i.e, the analysis in blood study with a relatively large sample size can detect meQTLs with relatively small effect size and low MAF, compared to brain and saliva studies. MeQTL detection in each study has been controlled for false positive.

If we restrict our analyses only to the meQTLs with consistent MAF across tissues (MAF difference <0.1 across tissues) and the effect sizes suggested by the power analyses (over 80%) for sample size n = 200 (the smallest one of the three datasets). The meQTL/target CpG overlapping ratios across tissues are shown in Fig.S2 with similar ranges to the report in Fig.1 (30~70%). However, fewer meQTLs, target CpGs and meQTL-CpG pairs are left after applying these restrictions. Given consistent results by applying a more conservative approach, the characteristics of overlapping meQTLs, leveraging maximum information of each study, are of value to the research community.


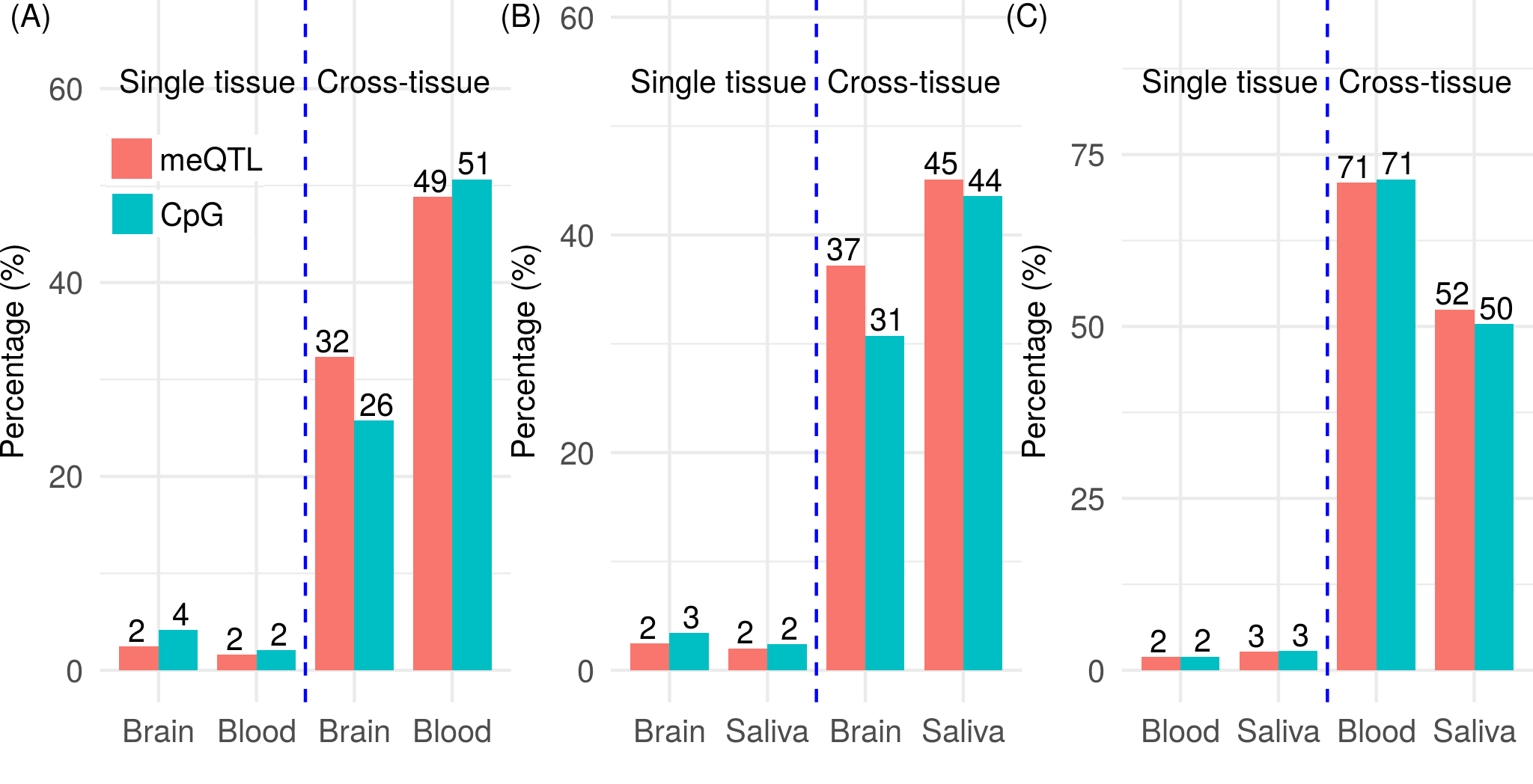


## Figure S2. The meQTL overlap between brain and blood (A), brain and saliva (B) and blood and saliva (C) by selecting the meQTLs with consistent MAF across tissues (MAF difference <0.1 across tissues) and the effect sizes to have power over 80% for 200 sample size.

**3) The relation between meQTL effect and Blood-brain correlation**


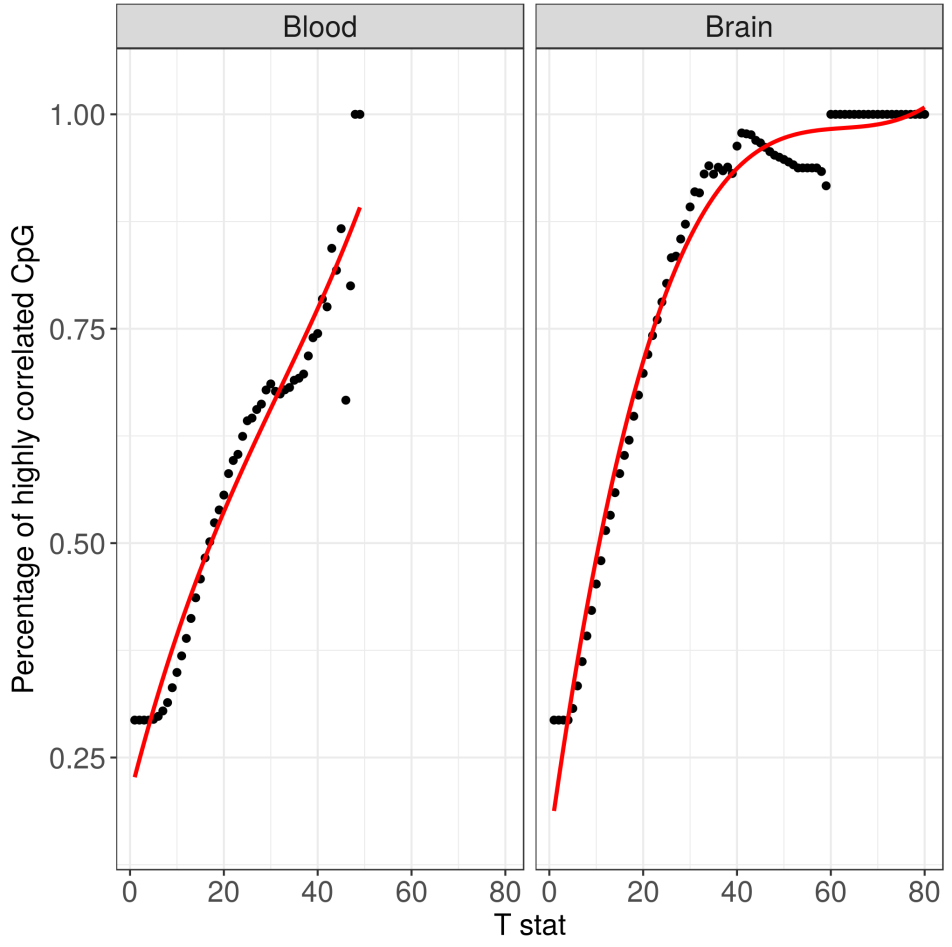


Figure S3. The relationship between meQTL effect(absolute value of T statistic) and the proportion of targeted CpGs which are highly correlated in brain and blood(r^2^>25%) over total target CpGs.

Table S1. The numbers of SNPs, CpGs, cis SNP-CpG pairs, meQTLs, and target CpGs in each tissue for analysis and their overlap across tissues

|  | SNPs | CpGs | Cis SNP-CpG (p≤1×10^-5^) | | meQTLs | Targeted CpGs |
| --- | --- | --- | --- | --- | --- | --- |
| Brain | 7,426,085 | 477,636 |  | 2,455,066 | 1,051,937 | 85,856 |
| Blood | 8,099,747 | 395,625 |  | 1,421,148 | 756,911 | 38,515 |
| Saliva | 3,622,550 | 363,366 |  | 466,953 | 287,247 | 26,752 |
| Brain vs. Blood | 5,824,390 | 395,083 | brain | 1,448,385 | 734,869 | 61,107 |
|  |  |  | blood | 1,188,404 | 633,938 | 36,604 |
|  |  |  | matched | 528,286 | 330,780 | 15,818 |
| Brain vs. Saliva | 3,284,823 | 363,352 | brain | 699,454 | 382,032 | 46,115 |
|  |  |  | saliva | 433,877 | 266,678 | 25,762 |
|  |  |  | matched | 212,435 | 143,566 | 14,065 |
| Blood vs. Saliva | 3,562,676 | 363,366 | blood | 598,922 | 343,842 | 29,314 |
|  |  |  | saliva | 460,616 | 283,383 | 26,141 |
|  |  |  | matched | 319,598 | 207,248 | 17,492 |
| Across all tissues | 3,258,095 | 363,352 | brain | 694,709 | 379,703 | 45,640 |
|  |  |  | blood | 564,150 | 323,600 | 28,790 |
|  |  |  | saliva | 430,956 | 264,914 | 25,478 |
|  |  |  | matched | 167,013 | 116,005 | 10,879 |

Table S2. Top 10 pathways involved by genes annotated from cross-tissue meQTL target CpGs in the pathway analysis

| Pathway Name | #Gene | P | FDR |
| --- | --- | --- | --- |
| Glycosaminoglycan biosynthesis | 8 | 3.74e-03 | 0.41 |
| HIF-1 signaling pathway | 34 | 3.99e-03 | 0.41 |
| Axon guidance | 52 | 7.16e-03 | 0.41 |
| Rap1 signaling pathway | 60 | 9.72e-03 | 0.41 |
| Propanoate metabolism | 13 | 1.07e-02 | 0.41 |
| MAPK signaling pathway | 70 | 1.17e-02 | 0.41 |
| Morphine addiction | 29 | 1.27e-02 | 0.41 |
| Focal adhesion | 57 | 1.36e-02 | 0.41 |
| Metabolic pathways | 301 | 1.36e-02 | 0.41 |
| GABAergic synapse | 28 | 1.45e-02 | 0.41 |

## References

1. Lin D, Calhoun VD, Wang Y-P: **Correspondence between fMRI and SNP data by group sparse canonical correlation analysis**. *Medical image analysis* 2014, **18**(6):891-902.
